# Development and Characterization of the Solvent-Assisted Active Loading Technology (SALT) for Liposomal Loading of Poorly Water-Soluble Compounds

**DOI:** 10.3390/pharmaceutics11090465

**Published:** 2019-09-09

**Authors:** Griffin Pauli, Wei-Lun Tang, Shyh-Dar Li

**Affiliations:** Faculty of Pharmaceutical Sciences, University of British Columbia, Vancouver, BC V6T 1Z3, Canada (G.P.) (W.-L.T.)

**Keywords:** liposome, water miscible solvents, remote loading, staurosporine, cancer, gambogic acid, loading gradients, mefloquine, child friendly formulation

## Abstract

A large proportion of pharmaceutical compounds exhibit poor water solubility, impacting their delivery. These compounds can be passively encapsulated in the lipid bilayer of liposomes to improve their water solubility, but the loading capacity and stability are poor, leading to burst drug leakage. The solvent-assisted active loading technology (SALT) was developed to promote active loading of poorly soluble drugs in the liposomal core to improve the encapsulation efficiency and formulation stability. By adding a small volume (~5 vol%) of a water miscible solvent to the liposomal loading mixture, we achieved complete, rapid loading of a range of poorly soluble compounds and attained a high drug-to-lipid ratio with stable drug retention. This led to improvements in the circulation half-life, tolerability, and efficacy profiles. In this mini-review, we summarize our results from three studies demonstrating that SALT is a robust and versatile platform to improve active loading of poorly water-soluble compounds. We have validated SALT as a tool for improving drug solubility, liposomal loading efficiency and retention, stability, palatability, and pharmacokinetics (PK), while retaining the ability of the compounds to exert pharmacological effects.

## 1. Introduction

### 1.1. Challenges in Delivery of Poorly Water-Soluble Drugs

Clinical translation of pharmaceutical compounds is often hindered by poor solubility. Over 40% of new chemical entities are not appreciably soluble in water, often resulting in limited therapeutic use, or abandonment as drug candidates [1,2]. Methods for improving apparent solubility include salt formulations, excipients, amorphous solid dispersions, pH adjustment, co-solvents, and nanocarriers [1,2]. There has been interest in the development of systematic approaches to overcoming low soluble compounds, as outlined in the developability classification system which describes a categorization of compounds based on permeability and solubility [3]. There has also been growing interest in the use of nanocarriers as a vehicle to overcome issues of solubility, as they serve as a platform technology and provide cell targeting [4]. Lipid-based nanocarriers (LNCs) represent a class of lipid particles, including solid lipid nanoparticles, micro- or nano-emulsions, polymer-lipid hybrid nanoparticles, and liposomes. 

#### 1.1.1. Liposomes and Drug Loading 

Liposomes are among the most studied class of LNCs for improving drug delivery [5]. Liposomes are nano-scale spheroid vesicles composed of one or multiple lipid bilayer(s) enclosing an aqueous core [5]. Liposomes can be manufactured using several methods, including thin-film hydration [6], reverse phase evaporation [7], and microfluidic mixing [8]. The process used to generate the liposome will impact its size and lamellarity [9,10,11]. Likewise, various lipids can be used to formulate liposomes with different properties, such as size, drug release kinetics, biocompatibility, surface charge, and cell targeting [12,13]. To encapsulate drugs into liposomes, two methods have been implemented; passive and active loading, both of which are briefly reviewed below. 

#### 1.1.2. Passive Loading

**Figure 1:**
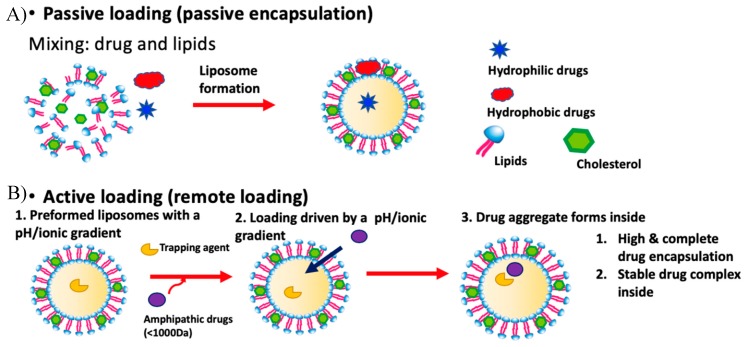
The two major methods for liposomal drug loading. (**A**) Passive loading involves co-current loading and liposomal formation. (**B**) In active loading, liposomes are formed containing a gradient used to load drugs.

Passive loading describes the procedure in which liposomes are formed concurrently with drug loading (Figure 1A). In general, hydrophilic compounds are distributed homogenously in the aqueous phase (both inside and outside the liposomes), whereas hydrophobic drugs are retained inside the lipid bilayer of liposomes, respectively. Specifically, when working with poorly water-soluble drugs, the drugs are first dissolved with lipids in an organic solvent, followed by solvent evaporation to prepare a drug containing thin film, which is later hydrated with an aqueous phase to prepare liposomes. When loading water-soluble drugs, the lipid film is dispersed in a drug-containing aqueous phase. 

#### 1.1.3. Limitations of Passive Loading 

The trapping efficiency of passive loading varies due to several factors, including drug solubility, vesicle size, lipid concentration, and preparation procedure. In most cases, the typical drug-to-lipid ratio (D/L) achieved by this passive loading technique is less than 0.05 (*w*/*w*) [14,15]. In addition, the entrapped drugs often cannot be retained stably due to weak association between the drugs and the liposomes, resulting in poor drug retention and storage stability. Passive loading often also results in a “burst release” phenomenon, whereby a large percentage of the entrapped drug is released quickly. 

#### 1.1.4. Active Loading

In active loading, liposomes are first generated containing a transmembrane gradient, i.e., the aqueous phases inside and outside the liposomes are different. Subsequently, an amphipathic drug dissolved in the exterior aqueous phase can permeate across the phospholipid bilayer(s), followed by interactions with a trapping agent in the core to lock-in the drug (Figure 1). In 1976, Deamer and Nicols [16,17,18] demonstrated that a pH gradient could be utilized to load catecholamine into liposomes, leading to stable retention in vitro. They formed liposomes in a low pH (pH 5) solution and then bathed the liposomes in an alkaline solution (pH 8) to create a 1000-fold difference in H_3_O^+^ ions across the bilayer. As the catecholamine molecules remained as the free form in the basic exterior, they freely permeated through the bilayer into the low pH core, where they were subsequently protonated. The charged catecholamine molecules were no longer membrane permeable and thus locked in. An accumulation of catecholamine molecules in the core of the liposomes was observed. Subsequent to the work of Dreamer et al. [18], Haran et al. [19] demonstrated that active loading can be achieved using an ammonium sulfate gradient. They produced liposomes with an interior containing ammonium sulfate. A concentrated solution of anthracycline was added to the liposomes and loading was then achieved after incubation at an elevated temperature. Anthracycline molecules formed aggregates in the core of the liposomes through precipitation with sulfate ions, and a high drug loading efficiency of >90% was achieved without burst drug leakage. Several other pH gradients have been established, including phosphate and calcium acetate [20]. Similar to the calcium acetate gradient, basified copper acetate gradients have also been employed for liposomal loading. 

#### 1.1.5. Limitations of Standard Active Loading 

Active loading remains a powerful tool that can be used to effectively and stably retain drugs in the core of liposomes. However, traditional active loading is predicated on compounds having high solubility and membrane permeability (i.e., amphipathic), so that they can be solubilized in the exterior aqueous phase and permeable through the lipid bilayer into the liposomal core. These selection criteria exclude a large number of hydrophobic drugs currently on the market or in the development pipeline. Excipients such as ß-cyclodextrin and super saturated solutions have been also used to increase the active loading efficiency of liposomes. These techniques have helped somewhat in overcome issues of poor solubility [21]. In addition, recent approaches have focused on examining the role of nanocrystillazaion inside liposomes to improve drug retention [22]. Although discussion of these techniques is beyond the scope of this paper, these methods have significant limitations, and consequently there is a need for an improved loading technology.

## 2. Solvent-Assisted Active Loading Technology (SALT)

### 2.1. Introduction

Our laboratory has developed an innovative technology for liposomal loading of poorly water-soluble drugs. Solvent-assisted active loading technology (SALT) allows for stable and efficient loading of poorly water-soluble drugs into the aqueous core of liposomes. SALT has demonstrated efficacy across a range of poorly soluble compounds and is a versatile method for drug loading. We hypothesized that including a small volume of water-miscible solvent in the loading mixture of liposomes and a hydrophobic drug could help solubilize the drug in the aqueous phase, increase the drug penetration through the liposomal bilayer and boost active loading encapsulation efficiency. pH adjustment is among the most commonly-used methods to increase drug solubility. When compared to pH adjustment, the SALT/liposome technique enhanced the solubility of our model drugs Staurosporine, Gambogic Acid and Mefloquine from 120 μg/mL to 1 mg/mL, 5 μg/mL to 1 mg/mL, and 0.6 mg/mL to 8 mg/mL, respectively [13,23,24].

### 2.2. Applications and Mechanism 

**Figure 2:**
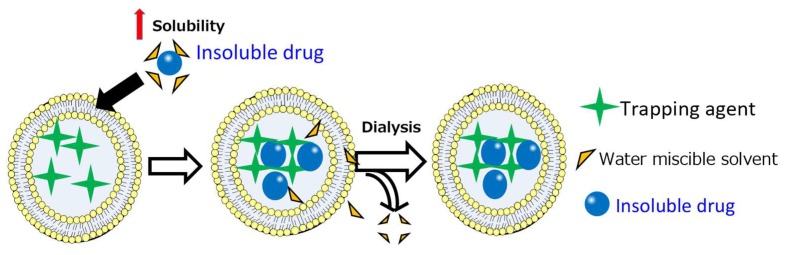
Solvent-assisted active loading technology (SALT) mechanism overview for liposomal loading of a poorly water-soluble drug.

A drug needs to be solubilized in the free form in the exterior phase of liposomes in order to effectively penetrate the lipid bilayer. Therefore, a small amount of solvent is included in the liposomal suspension for complete solubilisation of the compound. After penetration into the liposomal core, the drug can then interact with a trapping agent inside the liposomes to form insoluble precipitates for stable a “lock-in” effect. Finally, the solvent can be removed by dialysis or gel filtration (Figure 2). To test the feasibility of this idea, we performed proof-of-principle studies [13,23,24] with a range of poorly soluble drugs. We further characterized SALT through the use of various solvents, liposomal formulations and trapping agents to demonstrate its versatility. 

### 2.3. Proof-of-Principle with a Weak Base Drug

#### Staurosporine and Liposomal Loading

Staurosporine (STS) is a broadly acting alkaloid protein kinase inhibitor with demonstrated efficacy as an antitumor agent against several cancer types [25]. STS contains a secondary amine group, but its solubility remains negligible even at a low pH [26]. Despite promising in vitro results, STS development was hindered due to poor solubility and non-specificity [26]. We hypothesized that liposomal delivery of STS would minimize its systemic toxicity through enhanced tumor targeting while also overcoming issues surrounding poor solubility. Liposomal loading of STS was previously reported by Mukthavaram et al. [27]. They utilized an active loading strategy employing a reverse pH gradient [27]. However, they were only able to achieve 70% drug encapsulation and a low drug-to-lipid ratio (D/L) of 0.09 mol/mol [27]. We hypothesized that introducing a limited amount of dimethyl sulfoxide (DMSO) to the liposomal suspension could keep STS soluble during the active loading process and would also facilitate the permeation of the drug into the inner core of liposomes. We employed an ammonium sulfate gradient to effectively trap STS via sulfate-STS nano-aggregates inside the liposomal core and achieve 100% drug encapsulation at a high D/L of 0.31/1 (mol/mol). Subsequently, gel filtration facilitated the removal of DMSO generating a final liposomal product. In the following section, we report the SALT method optimization and results of PK and efficacy studies. 

**Liposomal Formulation:** A thin lipid film composed of 1,2-distearoyl-sn-glycero-3-phosphocholine (DSPC), cholesterol and PEGylated 1,2-distearoyl-sn-glycero-3-phosphoetholamine (DSPE-PEG2000) (55/40/5 mole ratio) (100 mg total lipids) was hydrated with 1 mL of 350 mM ammonium sulfate at 60 °C, followed by membrane extrusion to control the size ~100 nm with a polydispersity index (PDI) of <0.06. The liposomes were then dialyzed against an acetate buffer (100 mM, pH 5) to create a transmembrane gradient of ammonium sulfate.

**Figure 3:**
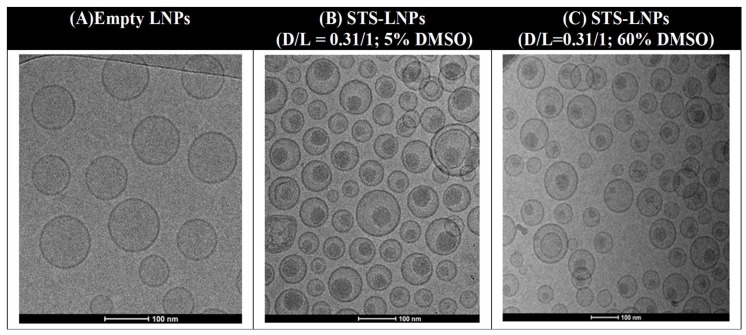
Cryo-TEM images of the drug free liposomes (**a**) and the staurosporine (STS)-Lipo loaded using 5% dimethyl sulfoxide (DMSO) (**b**) 60% DMSO (**c**). Scale bar represents 100 nm. Reprinted with permission from Tang et al., Pharmaceutical Research, published by Springer Nature, 2016 [24].

**Liposomal STS Loading Using SALT:** We dissolved STS in DMSO and added into the liposomal suspension at a range of D/L with a final DMSO content of 5–60% and incubated the mixture at room temperature or 60 °C. We found that at least 5% DMSO was required to achieve complete drug loading, and that complete drug loading was maintained over a range of DMSO concentrations between 5% and 60%. Loading kinetics was impacted by the temperature. In the presence of 5% DMSO, complete loading was achieved more rapidly (5 min) with incubation at 60 °C compared to room temperature (15 min). The highest D/L achieved for complete drug loading was 0.31 w/w. The size (~100 nm) and the PDI (<0.06) of the final STS-Lipo produced with various amounts of DMSO were comparable and remained unchanged compared to the empty liposomes. We also determined that STS leakage from the liposomes was minimal (<5%) after seven days incubation in 50% fetal bovine serum (FBS) at 37 °C. Finally, the cryo-transmission electron microscopy revealed that STS molecules form spherical precipitates inside the liposomal core (Figure 3). The data support the contention that STS was actively and stably loaded into the liposomal core by the SALT.

**Safety and Efficacy Studies:** It was found that the accumulated maximum tolerated dose (MTD) of STS-Lipo was 9 mg/kg relative to 3 mg/kg for free STS (dissolved in acetate buffer and 1% DMSO). STS-Lipo exhibited significantly improved efficacy against multidrug resistant EMT6-AR1 murine breast tumor in mice compared with free STS and docetaxel (DTX). The tumor growth was effectively impeded by STS-Lipo therapy and the average tumor volume was controlled by 150 mm^3^ in comparison to ~800 mm^3^ in the free STS group by day 18, while all tumors in the DTX and buffer treated mice all exceeded the endpoint size (1000 mm^3^) before day 14. The STS-Lipo treatment did not cause significant body weight loss. On the other hand, 3 out of 3 mice in the free STS group reached humane endpoints on day 9 due to the drug toxicity, and there was ~5% body weight loss in the mice treated with DTX. The data indicate that SALT enabled liposomal delivery of STS, leading to enhanced safety and efficacy compared to free STS and the standard taxane chemotherapy.

### 2.4. Proof-of-Principle with a Weak Acid Drug

#### 2.4.1. Introduction

After our initial discovery that SALT could promote loading of an insoluble weak base compound into the liposomal aqueous core to improve the drug delivery, we sought to further explore applications for SALT. We investigated whether SALT could efficiently load other drug classes. In addition, we examined whether solvents other than DMSO could be used in this technology and the role these solvents played in drug loading. To achieve this, we investigated the ability of SALT to improve loading of gambogic acid (GA). GA was selected as a model drug as it is water insoluble yet dissolvable in a range of water miscible solvents (>20 mg/mL). As such, we could investigate both the use of SALT on a different drug class (weak acid) and how SALT performs with solvents other than DMSO. 

#### 2.4.2. Gambogic Acid (GA)

GA is a naturally-derived compound found in traditional Asian medicines. GA has been reported to have anti-cancer and anti-inflammatory properties by acting through a number of cell processes [28,29,30]. Despite its promising results as an anti-cancer compound, its clinical development has been hindered by its poor water solubility (<5 µg/mL). Several methods have been attempted to overcome its issues of poor solubility, yet parameters relating to PK were only marginally improved [31,32,33,34,35]. We hypothesized that SALT could facilitate stable and efficient loading of GA into liposomes, leading to prolonged PK and tumor-targeted delivery.

**Figure 4:**
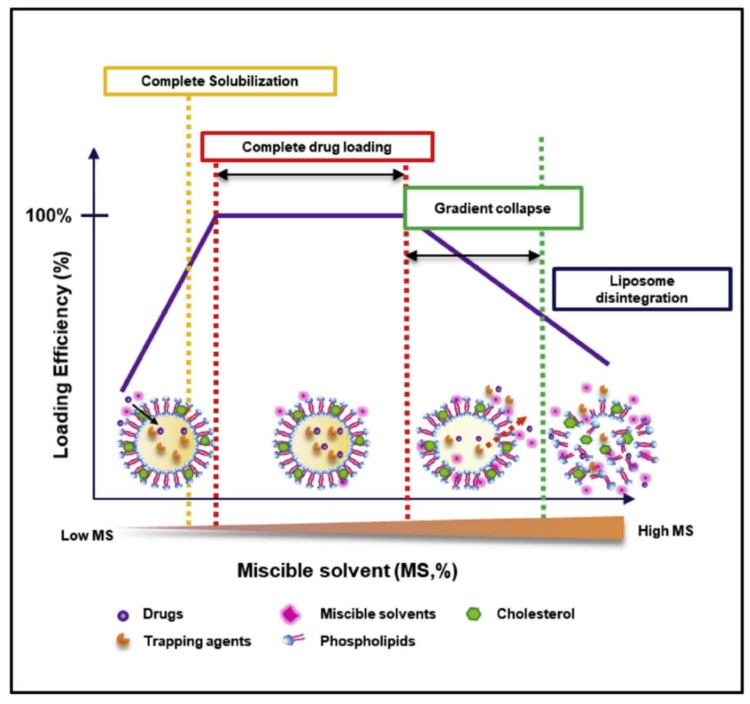
The solvent effect on drug loading in the SALT system. The figure is reprinted with permission from Tang et al., Biomaterials; published by Elsevier, 2018 [13].

**SALT Promotes GA Loading into Liposomes:** We began the GA study by seeking to find answers to three questions. Primarily, we were interested in determining whether SALT could be applied to load other drugs besides weak bases. Second, we sought to determine if other water miscible solvents were compatible with the SALT system. Third, we wanted to elucidate the mechanism behind the SALT system, specifically, to investigate the functions of solvents in promoting drug loading. GA is freely soluble in eight different water-miscible solvents; DMSO, DMF, EtOH, MeOH, acetonitrile, acetone, 1,4-dioxane, and NMP. We examined whether these solvents could be used to promote GA loading into liposomes. We prepared liposomes containing a gradient of basified copper acetate where [Cu^2+^]_interior_ > [Cu^2+^]_exterior_. We rationalized that after drug solubilization and loading into the liposomal core, GA would bind cooper via coordination complex. This drug-copper conjugate forms stable complexes and effectively traps GA in the liposome. This mechanism, which parallels other trapping strategies using compounds such as calcium acetate, helps boost drug retention and minimize leakage during storage. Our results showed that all 8 solvents could facilitate GA loading and the optimal solvent content was unique to the solvent. The data suggests the solvent function is twofold. Primarily, the solvent is used to completely dissolve the drug in the liposomal exterior water phase. This is required as large drug precipitates are impermeable to the lipid bilayer. However, we discovered that just complete solubilisation did not promote complete drug loading. After the solvent has dissolved the drug, additional solvent added serves to increase the permeability of the liposomal membrane (Figure 4). However, additional solvent must not exceed the limit that induces lipid membrane instability. In these studies, we have demonstrated that SALT is capable of loading both poorly soluble weak acid and base model drugs with high efficiency and retention. SALT is a versatile platform which could be used to load an array of compounds that may have been previously non-deliverable with liposomes. In addition to STS and GA, we further demonstrated that this method could be applied for loading other drugs, including artesunate, prednisolone hemisuccinate, and quercetin [13]. 

**Formulation Optimization:** After confirming that SALT could facilitate active loading of GA, we next sought to optimize the formulation by modifying the loading gradient and lipid composition to improve the D/L and drug retention. We studied GA loading efficiency in 1,2-distearoyl-sn-glycero-3-phosphocholine/cholesterol/1,2-distearoyl-sn-glycero-3-phosphoethanolamine-N-[amino(polyethyleneglycol)-2000 (DSPC/Chol/DSPE-PEG2K) liposomes with a range of transmembrane gradients, including magnesium gluconate, calcium formate, and copper acetate. All gradients achieved complete drug loading in the presence of 5 vol% DMSO at a D/L of 1/5 *w*/*w*. However, the basified copper acetate gradient (pH 9) demonstrated the highest drug retention in 50% serum: ~45% GA retained in the liposomes after 4 h incubation. We then modified the lipid composition to further enhance GA retention. Our results show that decreasing the cholesterol content from 45% to 0% increased GA retention from 40% to 68% after 2.5 h incubation in serum. We then compared formulations prepared with an unsaturated lipid 1,2-dioleoyl-sn-glycero-3-phosphocholine (DOPC) or a saturated lipid (DSPC): the DOPC-liposomes retained >95% of the drug after 24 h incubation in serum without significant size change. This finding was unanticipated as saturated liposomes containing lipids with a high transition temperature have been shown to increase doxorubicin retention relative to unsaturated lipids [36]. However, it has been discovered that unsaturated lipids, such as DOPC, may form flexible liposomes which trap hydrophobic molecules more effectively [37]. 

**Figure 5:**
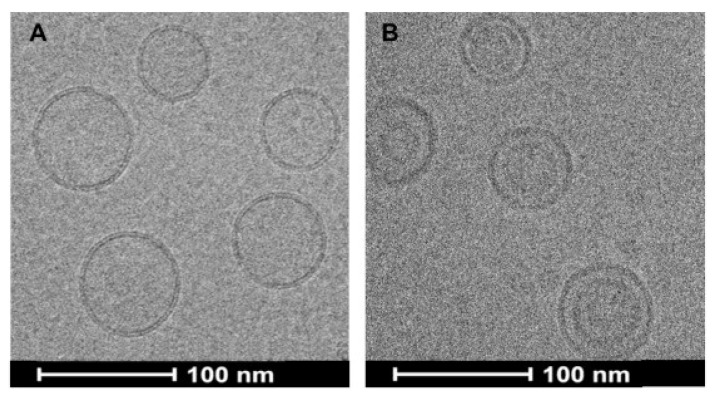
Cryo-TEM images of empty liposomes (DOPC/Chol/DSPE-PEG2K, 85/10/5 by mol%) (**A**) and Lipo-GA (**B**). The figure is reprinted with permission from Tang et al., Biomaterials; published by Elsevier, 2018 [13].

**Characterization of optimized Lipo-GA:** Transmission electron cryomicroscopy (CryoTEM) imaging revealed that liposomes displayed bi-lamellar structure with an electron dense core (Figure 5). Both these features have been previously reported with liposomes containing a copper gradient for drug loading [38,39], indicative of bilayer rearrangement and copper-GA complex formation in the core. The formation of copper-GA complexes, in addition to the optimized lipid composition resulted in stable retention of GA. 

**Safety, PK and Efficacy:** GA is known to induce apoptosis of red blood cells, leading to potential toxicity in vivo [40]. While free GA was highly hemolytic, the equivalent concentration of Lipo-GA showed no activity in inducing hemolysis. Compared to free GA, the Lipo-GA formulation resulted in an 18-fold increase in plasma half-life, a 20-fold higher mean residence time, a 7.5-fold higher area under the curve (AUC_0–∞_), and 10-fold decreased clearance, confirming its prolonged circulation relative to free GA. In two murine tumor models, we observed a significant dose-dependent reduction in tumour volume after Lipo-GA therapy. In particular, in the EMT6-AR1 multidrug resistant breast tumor model, one dose of Lipo-GA completely suppressed the tumor growth, while free GA only moderately inhibited 65% tumor growth. Mice treated with Lipo-GA showed no body weight loss, suggesting good safety.

## 3. Pediatric Formulation

We next explored a unique application for SALT. Malaria is the world’s leading parasitic disease [41], with over 200 million cases reported in 2015. Children under 5 years old are the most vulnerable population and account for over 70% of malaria associated deaths [42]. Treatment or prophylaxis of malaria often relies on the first line drug mefloquine (Mef) [43]. Despite its effectiveness as an antimalarial agent, Mef is difficult to accurately dose in children and neonates [44]. This is due to the lack of a pediatric formulation and Mef is typically administered by crushing up a portion of an adult tablet and administering with milk or food, in an effort to mask the extremely bitter taste. However, children often spit out the medicine, leading to subtherapeutic levels. Microemulsions of Mef have been developed to overcome poor solubility and increase bioavailability [45,46], yet their use in newborns is contradicted by risk of renal and liver damage caused by high concentrations of surfactants [47]. A pediatric pill of Mef is available (Artequin pediatric), yet this pill remains too large for neonatal dosing. We hypothesized that liposomal delivery of Mef could overcome many of these issues. Liposomal suspension of Mef could increase the solubility and subsequent absorption leading to higher bioavailability [48]. In addition, dosing the liquid suspension to children is easy and accurate. We hypothesized liposomes could also mask the taste of the bitter drug by shielding it from the taste buds. Liposomes can be lyophilized to prepare a powder formulation that is stable upon room temperature storage and orally dispersible for pediatric use. Lastly, liposomal formulations using only the neutral lipid DSPC and cholesterol can be considered very safe for oral administration in young children. The remaining challenge was how to prepare a stable Mef-Lipo formulation.

**Liposomal Loading of Mef Using SALT:** We first prepared liposomes (DSPC and cholesterol) containing an ammonium gradient, and then incubated the liposomes with Mef in the presence of 10% DMSO at a D/L concentration of 0.1 *w*/*w* at room temperature for 30 min to achieve complete loading. The liposomes were then purified by dialysis to remove DMSO and a formulation containing 8 mg/mL of Mef was obtained. 

**Lyophilizaition:** The Mef-Lipo suspension was subjected to freeze drying, aiming to prepare a solid powder formulation to facilitate longer storage time and produce a rapidly dissolvable formulation. To protect the integrity of the liposomes during lyophilization we buffered the exterior with 300 mM sucrose and 20 mM phosphate. Sucrose is a known lyoprotectant and phosphate was shown to increase the stability of liposomes [49]. The lyophilization process only increased the liposome size from 110 nm to 130 nm and the PDI remained <0.1. Importantly, we observed no significant drug leakage. The lyophilized liposomes containing Mef remained stable under the storage at room temperature for >3 months. In addition, they were rapidly dissolvable in water within 10 s, indicating an orally dispersible formulation. 

**Drug Release:** Both the liquid suspension and the lyophilized Mef-Lipo exhibited similar drug release profiles in simulated saliva, gastric, and intestinal fluids. In simulated saliva, both liquid and lyophilized Mef-Lipo demonstrated no observable drug release. Drug release was highest in the simulated stomach fluid for both formulations. There was no drug release in the simulated intestinal fluid in the absence of a bile salt for both preparations, while Mef was effectively released from the liposomes in simulated intestinal fluid supplemented with a bile salt. Our data indicate that Mef release from the liposomes was triggered by acid- or surfactant-induced destabilization of liposomes. The results also suggest that the liposomal formulation would effectively mask the drug taste in the oral cavity but rapidly release the drug in the gastrointestinal fluids. 

**Figure 6:**
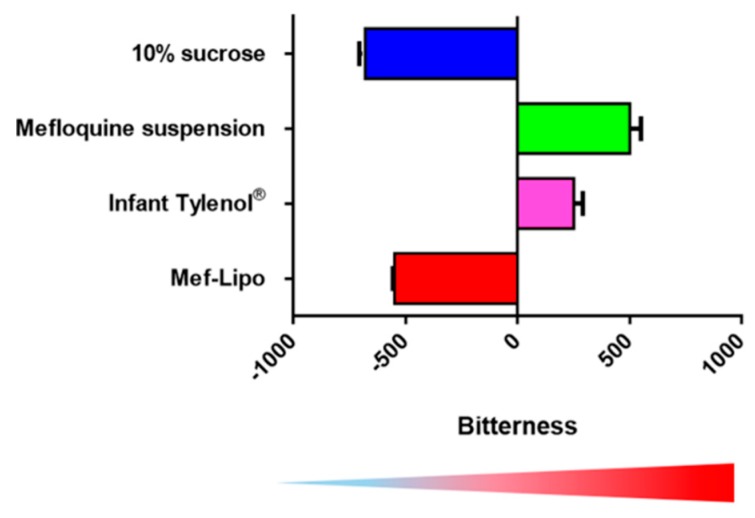
Quantification of bitterness of various drug formulations using the e-tongue. Data = mean ± Standard Deviation (SD) (*n* = 3). The figure is adapted with permission from Tang et al., Molecular Pharmaceutics; published by American Chemical Society, 2017 [23].

**Bitterness Masking:** To determine the bitterness masking effect of the liposomal technology, we employed the Astree e-tongue technology and compared the bitterness of Mef-Lipo to 10% sucrose, Mef suspension and Infant Tylenol^®^. As shown in Figure 6, the bitterness of the Mef-Lipo was similar to 10% sucrose indicating a palatable formulation, while the standard Mef suspension displayed high bitterness. 

**Pharmacokinetics and Bioavailability:** In PK and bioavailability (BA) studies in mice, our data confirmed that the liquid and lyophilized Mef-Lipo were comparable formulations with similar C_max_, T_max_, area under the curve (AUC), and BA (81−86%). Mef suspension, however, displayed ~20% decreased C_max_, AUC, and BA compared to the liposomal formulation. There was no difference in T_max_ and t_1/2_ among these three formulations, suggesting that the formulations did not alter the metabolism or elimination of the drug, and the difference in BA was due to the absorption. Liposomes might improve the absorption by preventing aggregation of Mef in the gastrointestinal tract, protectiing from degradation, and promoting direct uptake by enteric cells. 

## 4. Perspectives and Future Directions

We have demonstrated that SALT is a versatile loading technology for promoting the active loading of poorly water-soluble drugs into the liposomal core. We performed proof-of-principle studies with multiple drugs to characterize the versatility and robustness of SALT. Our studies show that SALT could improve drug solubility, liposomal loading efficiency, liposomal retention, stability, palatability, PK, and efficacy. SALT has the potential to overcome barriers currently impeding delivery of many compounds. We have also demonstrated applications of SALT in cancer therapy and child-friendly oral formulations. The application of SALT could extend beyond therapeutic drug loading and also be employed in the imaging and theranostic fields. Specifically, SALT could be useful in loading poorly soluble imaging probes into the core of liposomes. A frequent barrier to effective image-guided drug targeting is the weak association between the probe and the delivery vehicle [50]. SALT could help retain imaging probes inside the core of liposomes to improve their delivery to target tissues. Despite the high performance of SALT as a loading platform, there remain parameters that can be further optimized. As demonstrated in these specific studies, a limitation of SALT when implemented across different compounds is the requirement for optimization of the loading gradient, trapping method, and lipid composition for maximal loading efficiency and retention. However, trapping agents can be rationalized based on the drug structure and lipid composition can be empirically determined. In addition, a limitation of SALT is found in the need to find a water miscible solvent that can be used to load certain drugs, which may not always be possible. Moreover, some drugs may require large volumes of solvent that may result in degradation on the liposome itself prior to efficient drug loading although this was not found in our studies. The drugs themselves must almost possess an ionizable functional group, limiting potential candidates for this technology. Despite these limitations we anticipate that future innovations of SALT could help overcome loading of other species of drugs such as the poorly membrane-permeable yet water-soluble biomolecules such as proteins and nucleotides, both of which may benefit from liposomal delivery. SALT, however, is not limited to liposomes and will be implemented for drug loading in other bilayer-based delivery systems, further expanding the impact of this exciting technology. In addition, SALT can be used to improve current formulations that are prepared using passive loading. There exist several drugs on the market that are prepared using passive loading due to their hydrophobicity. SALT could be employed to re-formulate these existing liposomal products to improve the loading efficiency, stability and drug retention. SALT is a promising platform which may propel the field of lipid-based drug delivery to novel areas.

## References

[1] Savjani K.T., Gajjar A.K., Savjani J.K. (2012). Drug solubility: Importance and enhancement techniques. ISRN Pharm..

[2] Kalepu S., Nekkanti V. (2015). Insoluble drug delivery strategies: Review of recent advances and business prospects. Acta Pharm. Sin. B.

[3] Butler J.M., Dressman J.B. (2010). The developability classification system: Application of biopharmaceutics concepts to formulation development. J. Pharm. Sci..

[4] McNamara K., Tofail S.A.M. (2017). Nanoparticles in biomedical applications. Adv. Phys. X.

[5] Akbarzadeh A., Rezaei-Sadabady R., Davaran S., Joo S.W., Zarghami N., Hanifehpour Y., Samiei M., Kouhi M., Nejati-Koshki K. (2013). Liposome: Classification, preparation, and applications. Nanoscale Res. Lett..

[6] Zhang H. (2017). Thin-Film Hydration Followed by Extrusion Method for Liposome Preparation. Methods Mol. Biol..

[7] Cortesi R., Esposito E., Gambarin S., Telloli P., Menegatti E., Nastruzzi C. (1999). Preparation of liposomes by reverse-phase evaporation using alternative organic solvents. J. Microencapsul..

[8] Yu B., Lee R.J., Lee L.J. (2009). Microfluidic methods for production of liposomes. Methods Enzymol..

[9] Pattni B.S., Chupin V.V., Torchilin V.P. (2015). New Developments in Liposomal Drug Delivery. Chem. Rev..

[10] Maeki M., Kimura N., Sato Y., Harashima H., Tokeshi M. (2018). Advances in microfluidics for lipid nanoparticles and extracellular vesicles and applications in drug delivery systems. Adv. Drug Deliv. Rev..

[11] Dimov N., Kastner E., Hussain M., Perrie Y., Szita N. (2017). Formation and purification of tailored liposomes for drug delivery using a module-based micro continuous-flow system. Sci. Rep. UK.

[12] Anderson M., Omri A. (2004). The effect of different lipid components on the in vitro stability and release kinetics of liposome formulations. Drug Deliv..

[13] Tang W.L., Tang W.H., Szeitz A., Kulkarni J., Cullis P., Li S.D. (2018). Systemic study of solvent-assisted active loading of gambogic acid into liposomes and its formulation optimization for improved delivery. Biomaterials.

[14] Gubernator J. (2011). Active methods of drug loading into liposomes: Recent strategies for stable drug entrapment and increased in vivo activity. Expert Opin. Drug Deliv..

[15] Zhao Y.C., May J.P., Chen I.W., Undzys E., Li S.D. (2015). A Study of Liposomal Formulations to Improve the Delivery of Aquated Cisplatin to a Multidrug Resistant Tumor. Pharm. Res..

[16] Bally M.B., Mayer L.D., Loughrey H., Redelmeier T., Madden T.D., Wong K., Harrigan P.R., Hope M.J., Cullis P.R. (1988). Dopamine accumulation in large unilamellar vesicle systems induced by transmembrane ion gradients. Chem. Phys. Lipids.

[17] Mayer L.D., Bally M.B., Cullis P.R. (1986). Uptake of Adriamycin into Large Unilamellar Vesicles in Response to a Ph Gradient. Biochim. Biophys. Acta.

[18] Deamer D.W., Prince R.C., Crofts A.R. (1972). The response of fluorescent amines to pH gradients across liposome membranes. Biochim. Biophys. Acta.

[19] Haran G., Cohen R., Bar L.K., Barenholz Y. (1993). Transmembrane Ammonium-Sulfate Gradients in Liposomes Produce Efficient and Stable Entrapment of Amphipathic Weak Bases. Biochim. Biophys. Acta.

[20] Clerc S., Barenholz Y. (1995). Loading of amphipathic weak acids into liposomes in response to transmembrane calcium acetate gradients. Biochim. Biophys. Acta.

[21] Bhatt P., Lalani R., Vhora I., Patil S., Amrutiya J., Misra A., Mashru R. (2018). Liposomes encapsulating native and cyclodextrin enclosed paclitaxel: Enhanced loading efficiency and its pharmacokinetic evaluation. Int. J. Pharm..

[22] Li T., Cipolla D., Rades T., Boyd B.J. (2018). Drug nanocrystallisation within liposomes. J. Control. Release.

[23] Tang W.L., Tang W.H., Chen W.C., Diako C., Ross C.F., Li S.D. (2017). Development of a Rapidly Dissolvable Oral Pediatric Formulation for Mefloquine Using Liposomes. Mol. Pharm..

[24] Tang W.L., Chen W.C., Roy A., Undzys E., Li S.D. (2016). A Simple and Improved Active Loading Method to Efficiently Encapsulate Staurosporine into Lipid-Based Nanoparticles for Enhanced Therapy of Multidrug Resistant Cancer. Pharm. Res..

[25] Schwartz G.K., Redwood S.M., Ohnuma T., Holland J.F., Droller M.J., Liu B.C.S. (1990). Inhibition of Invasion of Invasive Human Bladder-Carcinoma Cells by Protein-Kinase-C Inhibitor Staurosporine. J. Natl. Cancer Inst..

[26] Akinaga S., Gomi K., Morimoto M., Tamaoki T., Okabe M. (1991). Antitumor-Activity of Ucn-01, a Selective Inhibitor of Protein-Kinase-C, in Murine and Human Tumor-Models. Cancer Res..

[27] Mukthavaram R., Jiang P.F., Saklecha R., Simberg D., Bharati I.S., Nomura N., Chao Y., Pastorino S., Pingle S.C., Fogal V. (2013). High-efficiency liposomal encapsulation of a tyrosine kinase inhibitor leads to improved in vivo toxicity and tumor response profile. Int. J. Nanomed..

[28] Wu Z.Q., Guo Q.L., You Q.D., Zhao L., Gu H.Y. (2004). Gambogic acid inhibits proliferation of human lung carcinoma SPC-A1 cells in vivo and in vitro and represses telomerase activity and telomerase reverse transcriptase mRNA expression in the cells. Biol. Pharm. Bull..

[29] Li X.F., Liu S.T., Huang H.B., Liu N.N., Zhao C., Liao S.Y., Yang C.S., Liu Y.R., Zhao C.G., Li S.J. (2013). Gambogic Acid Is a Tissue-Specific Proteasome Inhibitor In Vitro and In Vivo. Cell Rep..

[30] Ishaq M., Khan M.A., Sharma K., Sharma G., Dutta R.K., Majumdar S. (2014). Gambogic acid induced oxidative stress dependent caspase activation regulates both apoptosis and autophagy by targeting various key molecules (NF-kappa B, Beclin-1, p62 and NBR1) in human bladder cancer cells. Biochim. Biophys. Acta.

[31] Cai L.L., Qiu N., Xiang M.L., Tong R.S., Yan J.F., He L., Shi J.Y., Chen T., Wen J.L., Wang W.W. (2014). Improving aqueous solubility and antitumor effects by nanosized gambogic acid-mPEG(2000) micelles. Int. J. Nanomed..

[32] Doddapaneni R., Patel K., Owaid I.H., Singh M. (2016). Tumor neovasculature-targeted cationic PEGylated liposomes of gambogic acid for the treatment of triple-negative breast cancer. Drug Deliv..

[33] Zhang Z., Qian H.Q., Yang M., Li R.T., Hu J., Li L., Yu L.X., Liu B.R., Qian X.P. (2017). Gambogic acid-loaded biomimetic nanoparticles in colorectal cancer treatment. Int. J. Nanomed..

[34] Yin D.K., Yang Y., Cai H.X., Wang F., Peng D.Y., He L.Q. (2014). Gambogic Acid-Loaded Electrosprayed Particles for Site-Specific Treatment of Hepatocellular Carcinoma. Mol. Pharm..

[35] Zhang D.H., Zou Z.Y., Ren W., Qian H.Q., Cheng Q.F., Ji L.L., Liu B.R., Liu Q. (2018). Gambogic acid-loaded PEG-PCL nanoparticles act as an effective antitumor agent against gastric cancer. Pharm. Dev. Technol..

[36] Charrois G.J.R., Allen T.M. (2004). Drug release rate influences the pharmacokinetics, biodistribution, therapeutic activity, and toxicity of pegylated liposomal doxorubicin formulations in murine breast cancer. Biochim. Biophys. Acta.

[37] Chang H.I., Yeh M.K. (2012). Clinical development of liposome-based drugs: Formulation, characterization, and therapeutic efficacy. Int. J. Nanomed..

[38] Kheirolomoom A., Mahakian L.M., Lai C.Y., Lindfors H.A., Seo J.W., Paoli E.E., Watson K.D., Haynam E.M., Ingham E.S., Xing L. (2010). Copper-Doxorubicin as a Nanoparticle Cargo Retains Efficacy with Minimal Toxicity. Mol. Pharm..

[39] Dicko A., Kwak S., Frazier A.A., Mayer L.D., Liboiron B.D. (2010). Biophysical characterization of a liposomal formulation of cytarabine and daunorubeticin. Int. J. Pharm..

[40] Lupescu A., Jilani K., Zelenak C., Zbidah M., Shaik N., Lang F. (2012). Induction of Programmed Erythrocyte Death by Gambogic Acid. Cell. Physiol. Biochem..

[41] Breman J.G., Alilio M.S., Mills A. (2004). Conquering the intolerable burden of malaria: What’s new, what’s needed: A summary. Am. J. Trop. Med. Hyg..

[42] Caminade C., Kovats S., Rocklov J., Tompkins A.M., Morse A.P., Colon-Gonzalez F.J., Stenlund H., Martens P., Lloyd S.J. (2014). Impact of climate change on global malaria distribution. Proc. Natl. Acad. Sci. USA.

[43] Schlagenhauf P., Adamcova M., Regep L., Schaerer M.T., Bansod S., Rhein H.G. (2011). Use of mefloquine in children—A review of dosage, pharmacokinetics and tolerability data. Malar. J..

[44] White N.J. (2004). Antimalarial drug resistance. J. Clin. Investig..

[45] du Plessis L.H., Helena C., van Huysteen E., Wiesner L., Kotze A.F. (2014). Formulation and evaluation of Pheroid vesicles containing mefloquine for the treatment of malaria. J. Pharm. Pharmcol..

[46] Mbela T.K.M., Deharo E., Haemers A., Ludwig A. (1998). Submicron oil-in-water emulsion formulations for mefloquine and halofantrine: Effect of electric-charge inducers on antimalarial activity in mice. J. Pharm. Pharmacol..

[47] Schwartzberg L.S., Navari R.M. (2018). Safety of Polysorbate 80 in the Oncology Setting. Adv. Ther..

[48] Daeihamed M., Dadashzadeh S., Haeri A., Akhlaghi M.F. (2017). Potential of Liposomes for Enhancement of Oral Drug Absorption. Curr. Drug Deliv..

[49] Kannan V., Balabathula P., Thoma L.A., Wood G.C. (2015). Effect of sucrose as a lyoprotectant on the integrity of paclitaxel-loaded liposomes during lyophilization. J. Liposome Res..

[50] Tang W.L., Tang W.H., Li S.D. (2018). Cancer theranostic applications of lipid-based nanoparticles. Drug Discov. Today.

